# CX3CL1 Pathway as a Molecular Target for Treatment Strategies in Alzheimer’s Disease

**DOI:** 10.3390/ijms24098230

**Published:** 2023-05-04

**Authors:** Giulia Bivona, Matilda Iemmolo, Giulio Ghersi

**Affiliations:** 1 Department of Biomedicine, Neurosciences and Advanced Diagnostics, University of Palermo, 90127 Palermo, Italy; 2 Department of Biological, Chemical and Pharmaceutical Sciences and Technologies (STEBICEF), University of Palermo, Viale delle Scienze, Ed. 16, 90128 Palermo, Italy

**Keywords:** Alzheimer’s disease, pathogenesis, microglia, CX3CL1, neuron-to-glia communication, fractalkine, β-amyloid, central nervous system

## Abstract

Alzheimer’s disease (AD) is a scourge for patients, caregivers and healthcare professionals due to the progressive character of the disease and the lack of effective treatments. AD is considered a proteinopathy, which means that aetiological and clinical features of AD have been linked to the deposition of amyloid β (Aβ) and hyperphosphorylated tau protein aggregates throughout the brain, with Aβ and hyperphosphorylated tau representing classical AD hallmarks. However, some other putative mechanisms underlying the pathogenesis of the disease have been proposed, including inflammation in the brain, microglia activation, impaired hippocampus neurogenesis and alterations in the production and release of neurotrophic factors. Among all, microglia activation and chronic inflammation in the brain gained some attention, with researchers worldwide wondering whether it is possible to prevent and stop, respectively, the onset and progression of the disease by modulating microglia phenotypes. The following key points have been established so far: (i) Aβ deposition in brain parenchyma represents repeated stimulus determining chronic activation of microglia; (ii) chronic activation and priming of microglia make these cells lose neuroprotective functions and favour damage and loss of neurons; (iii) quiescent status of microglia at baseline prevents chronic activation and priming, meaning that the more microglia are quiescent, the less they become neurotoxic. Many molecules are known to modulate the quiescent baseline state of microglia, attracting huge interest among scientists as to whether these molecules could be used as valuable targets in AD treatment. The downside of the coin came early with the observation that quiescent microglia do not display phagocytic ability, being unable to clear Aβ deposits since phagocytosis is crucial for Aβ clearance efficacy. A possible solution for this issue could be found in the modulation of microglia status at baseline, which could help maintain both neuroprotective features and phagocytic ability at the same time. Among the molecules known to influence the baseline status of microglia, C-X3-chemokine Ligand 1 (CX3CL1), also known as Fractalkine (FKN), is one of the most investigated. FKN and its microglial receptor CX3CR1 are crucial players in the interplay between neurons and microglia, modulating the operation of some neural circuits and the efficacy and persistence of immune response against injury. In addition, CX3CL1 regulates synaptic pruning and plasticity in the developmental age and in adulthood, when it strongly impacts the hippocampus neurogenesis of the adult. CX3CL1 has an effect on Aβ clearance and tau phosphorylation, as well as in microglia activation and priming. For all the above, CX3CL1/CX3CR1 signalling has been widely studied in relation to AD pathogenesis, and its biochemical pathway could hide molecular targets for novel treatment strategies in AD. This review summarizes the possible role of CX3CL1 in AD pathogenesis and its use as a potential target for AD treatment.

## 1. Introduction

Alzheimer’s disease (AD) is the most common cause of dementia, representing a fatal and burdening disease without an effective treatment and with a high mortality rate [1].

AD affects more than 50 million people worldwide [2]. A decrease in dementia incidence in high-income countries has been posited [3], although a scientific controversy has partially disappointed that expectation [4].

AD can be distinguished into sporadic and familial type, with sporadic AD being the most frequent form of the disease. Major risk factors for developing the disease are age > 65 years and carrying the APOEε4 allele, even if many other risk factors, counting genetics and lifestyle-associated risks, have been identified [5].

AD clinical presentation includes cognitive impairment with memory loss, language issues and neuro-behavioural symptoms. These symptoms can evolve from the preclinical stage of the disease to the overt presentation, covering a time span of some years, within the so-called “AD continuum” [1].

Established disease hallmarks are the amyloid β (Aβ) and hyperphosphorylated tau aggregates. Aβ is generated through the cleavage of the amyloid precursor protein (APP) by β and γ secretase enzymes, producing different Aβ peptides. These can bind to one another, forming Aβ oligomers, polymers and plaques, with the oligomers representing the most neurotoxic compounds [6]. Tau protein normally provides for the stabilization of microtubules in neurons; pathologic alterations of tau, such as hyperphosphorylation, lead to dysfunctional protein with microtubule detachment [7]. Tau aggregates are deemed to be more toxic towards neurons compared to Aβ deposits [8].

From a pathogenic perspective, the “amyloid cascade hypothesis” of AD postulates that neurodegeneration during AD is subsequent to Aβ deposition. However, it has been reported that decreasing Aβ load does not improve cognitive symptoms in AD models and patients and that Aβ-targeting therapies do not impact disease progression [9]. Hence, the amyloid cascade hypothesis has become insufficient to explain AD pathogenesis, and a new path to be tracked on this issue has been found in microglia [10].

Microglia, the only mesenchymal-origin brain cells, are resident innate immune cells belonging to the myeloid lineage. They derive from yolk sac progenitors and are able to reproduce, regardless of bone marrow haematopoiesis, through a self-renewal mechanism [11]. Microglia display several functions, including surveillance of the brain microenvironment, central nervous system (CNS) homeostasis regulation and immune response against injury.

Under physiological conditions, microglia have highly ramified processes that continuously retract and extend themselves to contact neurons and astrocytes. This extra-synaptic, bidirectional dialogue between neurons and microglia is essential for brain function and protection against injury [12]. Neuron-to-microglia communication depends on many signalling pathways and molecules, including CD200, CD22, CD47 and CX3CL1, with CX3CL1 being crucial [13,14]. Shortly, neuron-to-microglia crosstalk has two main purposes: (i) maintaining microglia in an anti-inflammatory, quiescent state, and (ii) modulating synaptic plasticity of some circuits (e.g., hippocampus GABAergic neural circuits) [15,16,17,18]. Keeping microglia quiescent and sustaining synaptic plasticity are both relevant for normal function in healthy brains since quiescent microglia guarantee an anti-inflammatory milieu, protecting from inflammation-based pathologies [19]. In addition, maintaining an anti-inflammatory microenvironment in the brain leads to neuroprotection, as documented by studies showing that chronic activation of microglia and pro-inflammatory conditions can cause neuronal loss and death [1,9,20,21]. On the other hand, synaptic plasticity allows for the growth and activity of neural networks, being essential for circuit wiring, operation and strength. Relating to these cognitive functions, it should be noted that promoting an anti-inflammatory microenvironment in the brain and sustaining synaptic plasticity is fundamental for the adult neurogenesis of the hippocampus and learning and memory processes [22]. Indeed, an anti-inflammatory microenvironment is necessary to protect the neuronal progenitor cells (NPSs) of the subgranular zone and subventricular zone of the hippocampus, forming the so-called neurogenic niche [23,24]. After several stages of differentiation and maturation, newborn neurons are included within hippocampal circuits and carry on learning and memory processes as well as older neurons do [25]. It has been reported that neurogenesis impairment is strongly associated with AD pathogenesis and even precedes the appearance of characteristic features of the disease, such as amyloid and tau aggregates [25,26].

Given that neuron-to-microglia crosstalk and CX3CL1 sustain core functions involved in AD pathogenesis, such as hippocampal neurogenesis, memory and learning processes and quiescent microglia status [27], several studies investigated the possible role of the CX3CL1 signalling pathway in AD onset and progression, to clarify whether the chemokine, or some molecules belonging to its signalling pathway, can be considered as a valuable target for AD treatment strategies.

This review summarizes the possible role of CX3CL1 in AD pathogenesis and its use as a potential target for AD treatment.

## 2. CX3CL1

CX3CL1 belongs to the CX3C family of chemokines, which are subtyped into four families (C, CC, CXC, CX3C) based on their biochemical structure, with two cysteine residues that can or cannot be separated by amino acids. The CX3C family is characterized by the presence of three amino acids between the cysteine residues and includes CX3CL1, also known as fractalkine (FKN). Generally, chemokines are expressed throughout the brain, where they mediate the interaction between neurons and glial cells, along with immune and chemotactic activities [28,29].

FKN is a CX3C chemokine existing in the transmembrane and as a soluble isoform. The former consists of an N-terminal 77 amino acid domain, a 241 amino acid mucin-like stalk and an 18 amino acid transmembrane region and a 37 C-terminal domain. Soluble FKN consists of the N-terminal chemokine domain and results from the cleavage of a disintegrin and metalloproteases (ADAM) 10, ADAM 17 and cathepsin S, at the site of the mucin-like stalk [9,30,31]. β secretase (BACE) carries on FKN cleavage as well, as recently demonstrated by Fan et al. [32]. It is widely accepted that the membrane-bound form acts as an adhesion molecule while the soluble form exerts neuromodulation signalling activity [33,34]. Once the soluble form is generated, it interacts with CX3CR1, the sole FKN receptor that is found to be mostly present in microglia and partly expressed by astrocytes and neurons [35]. CX3CR1 is a G protein-coupled receptor, and its downstream signalling pathway is the main connection route between neurons and microglia [36,37]. Many molecules acting as CX3CR1 signal downstream transducers upon FKN binding are known [23,32,36,38,39,40,41,42] (Table 1). Describing the isoforms of FKN and the mechanisms to generate them is of particular interest when considering that differential activity between soluble and membrane-bound proteins has been reported. For instance, it has been documented that soluble FKN is beneficial in rescuing memory, whereas the membrane-bound isoform is not [43]. Therefore, proteolytic cleavage of CX3CL1 seems to be crucial for the regulation of FKN activity in the brain, both in healthy and pathologic conditions [13].

Hippocampus and cortex neurons highly express CX3CL1 [44,45], with the chemokine exerting neuroprotective and neurotrophic effects. CX3CL1 neuroprotection mainly depends on the reduction in microglial activation and inhibition of pro-inflammatory gene expression and cytokines (IL-1 β, IL-6, TNF-α) synthesis [46]. In physiological conditions, the CX3CL1 signal has been shown to influence cognitive function as measured by Morris water maze deficit and contextual fear conditioning, along with motor learning and hippocampal neurogenesis [22]. Notably, administration of anIL-1β receptor antagonist is able to reverse detrimental effects on cognition, suggesting that impairment in cognitive function associated with CX3CL1 deficiency is dependent on IL-1β production and activity [22]. In 2015, Febinger et al., demonstrated that altering FKN signalling leads to a dysfunctional microglial response and subsequent neuronal damage [47]. The neurotrophic effect of the FKN pathway is associated with the capability of the CX3CL1/CX3CR1 axis to regulate synaptic plasticity, with great influence on the neural circuit’s connectivity [30,33,46]. Studies in animal models have shown that CX3CL1 is overexpressed during memory-associated synaptic plasticity [48], and CX3CL1 deficient mice display altered synaptic pruning [49]. Several mechanisms have been proposed to explain the impact of FKN signalling in synaptic plasticity, including the regulation of long-term potentiation (LTP), nitric oxide (NO) signalling and the production of brain-derived neurotrophic factor (BDNF) [50]. Further, CX3CL1 deficient mice have been reported to display impaired neurogenesis [24] (Table 2).

### 2.1. Limitations of the Studies on the Role of the CX3CL1/CX3CR1 Axis in AD Pathogenesis 

Given all the above, the role of CX3CL1 in in vivo and in vitro AD models has been investigated, achieving controversial results. It can be stated that discrepancies among the findings obtained depend on several factors. Firstly, regarding the in vivo studies using different AD animal models, the typology of mice utilized has been found to sharply influence the findings obtained. In general, suppressing CX3CL1 signalling in Aβ pathology models (i.e., hAPP, APP-PS1 and CRND8 mice) and tau pathology models (hTau mice) gives rise to opposite results due to a Janus behaviour of CX3CL1 toward Aβ and tau aggregate deposition [39,40]. Indeed, the chemokine signal normally reduces the amyloid clearance (by inhibiting microglial phagocytosis), favouring amyloid aggregate deposition, but avoids tau phosphorylation, protecting from neurofibrillary tangle accumulation [40,51,52]. Consequently, suppressing the CX3CL1 signal in Aβ pathology mouse models results in ameliorating Aβ burden, but it does worsen tau phosphorylation and exacerbates cognitive deficits in tau-pathology mouse models [19,46]. In addition, differential expression of CX3CR1 throughout the brain influences Aβ deposition and toxicity [53]. Another main flaw affecting the studies on this topic is that detailed knowledge about different soluble versus membrane-bound FKN isoform functions is lacking. This increased discrepancies and confounders among the experiments and findings and prompted some authors to investigate the most common commercial peptides used as FKN reagents in scientific research from a pharmacologic point of view [31]. Interestingly, the authors concluded that findings from the studies on FKN in neurodegenerative diseases should be taken with a grain of salt and great caution is needed in selecting peptides as well.

Another main factor influencing the unidirectionality of the results is using animal models per se. AD mouse models often present transgenic tau overexpression that leads to a forceful, extreme inflammation, artificially altering experimental processes and results, compared to the milder inflammatory response by microglia that is more likely to occur in the brain, especially in the early stage of disease [54,55,56]. This advantage should be added to the most known one, which is the simplified nature of in vivo models compared to humans, resulting in possible difficulties in translating findings and conclusions from preclinical to clinical models. Finally, the administration route of some molecules that have been found to be either potential targets for treatment or stimulating factors for some pathways involved in the disease pathogenesis (as is the case for CX3CL1) remains a challenging issue. Stereotactic injections, viral vectors and the use of mesenchymal stem cell-derived products represent the most common methods for molecule administration among AD studies experiments, calling attention to the need to identify novel strategies for administering molecules of interest [9].

That said, the main findings from the studies addressing a possible role for CX3CL1 in AD pathogenesis and, most importantly, as a target for AD treatment can be summarized as follows.

### 2.2. In Vitro Studies

Recently, Guo et al., investigated the effect of bone marrow mesenchymal stem cell (BMSC)-derived CX3CL1 on SH-SY5Y neuroblastoma cells under Aβ1-42 inducement. They found that BMSC-derived CX3CL1 inhibited cell damage induced by Aβ1-42, as defined by axonal length, cell growth and synaptic protein expressions. In addition, the authors described the putative mechanism underlying their results, that is, the TXNIP/NLRP3 pathway [42]. However, the authors pointed out that the experiments were insufficient to explain either the involvement of BMSC-derived CX3CL1 axis in brain inflammation or which CX3CL1 isoform, between the membrane-bound and the soluble ones, was responsible for reducing Aβ1-42-induced injury in SH-SY5Y cells.

Dworzak et al., performed complex analyses, including both in vivo and in vitro AD models, starting from the assumption that CX3CR1 can be expressed by neurons and microglia in some contexts, having different influences on the local response to Aβ deposition. Examination of differences between neuronal and microglial CX3CR1 was defined as synaptic toxicity and dysfunction induced by Aβ deposition. Using primary cultures of CX3CR1-/- neurons and microglia, the authors demonstrated that Aβ peptide composition and conformation affects synaptic transmission per se, and that neuronal CX3CR1 is more present within the hippocampus compared to the cortex, making its deficiency protective toward neurotoxicity. The authors suggested that hippocampal neurons are more prone to Aβ toxicity due to the high, selective presence of CX3CR1 in this brain area [53]. However, considering the controversy around the presence of CX3CR1 on neurons, with some authors [35,57,58] reporting selective microglial expression of the receptor, Dworzak’s findings should be interpreted with caution.

Bolos et al., examined whether microglia could carry out tau internalization through a CX3CR1-mediated mechanism, by treating primary cultures from the cerebral cortex of 2-day-old mice with phosphoTau Cy5 and control Cy5. Immunofluorescence analysis showed that CX3CR1 mediates tau internalization by microglia, as measured by calculating the amount of Cy5 positive area within microglial cells [59].

### 2.3. In Vivo Studies

At the beginning of the investigations on the CX3CL1/CX3CR1 axis in AD, the focus has been the CX3CR1 receptor for a long time, with many studies performed using CX3CR1 deficient AD mouse models [38,39,40,59]. Lee et al., reported CX3CR1 deficiency reduced Aβ deposition and neuronal loss in APP-PS1 and R1.40 mouse models of AD, along with the reduction in microglia surrounding Aβ plaques, as demonstrated by immunohistochemical analysis using the CD68 microglial marker [40]. The experiments of Lee et al., contributed to confirming much information about FKN, such as its capability of modulating inflammation in the brain and the regulation of phagocytosis activity in microglia, that nowadays are fully established. Bhaskar et al., reported CX3CR1 deficiency worsened tau phosphorylation and brain inflammation in the tau-pathology mouse model of AD hTau mice [39], confirming that different experiments give rise to different effects, either beneficial or detrimental, depending on the mouse model used. Successive experiments have focused on the role of FKN signalling regardless of the CX3CR1 receptor. In 2019, Fan et al., examined the role of the CX3CL1 C-terminal fragment (CX3CL1ct), which is the intracellular domain that is produced after two sequential cleavages of the transmembrane chemokine by BACE and γ secretase [32]. The experiments were performed by generating the mouse models of AD 5xFAD, in which amyloid deposition and neuronal loss were found to diminish significantly due to increased neurogenesis. Authors concluded that overexpression of CX3CL1ct enhances SGZ and SVZ neurogenesis via activation of transcriptional factors of some genes involved in neurogenesis, with TGFβ3, bone morphogenetic protein (BMP) and Smad being the most implicated pathways. Importantly, the authors described the back-signalling mechanism through which CX3CL1ct is translocated in the nucleus, where it induces the regulation of the above-mentioned transcription factors. The importance of these results is that the authors proved the beneficial effect of the chemokine in AD models and showed stimulation of neurogenesis to be independent of the receptor CX3CR1, which moved the focus of investigations from neuron-to-glia communication signalling pathways toward the expression levels of BACE in neurons and the production of the sole chemokine C-terminal fragment in these cells [32]. Subsequent analysis of Fan et al., was carried out using transgenic Tg mice overexpressing CX3CLK1 and a PS19 AD mouse model, confirming that FKN enhances neurogenesis via Smad signalling and showing that the CX3CL1ct reduces neuronal loss and improves cognitive function, as defined by learning and memory process, in AD mice [60].

Hickman et al., analyzed PS1-APP mice heterozygous for CX3CR1 (PS1-APP-CX3CR1+/−), documenting that CX3CR1 deficiency is associated with the reduction in Aβ levels, plaque burden and improved cognitive function [61]. Due to the ability of CX3CL1 to enhance the production of some molecules involved in Aβ degradation, the authors suggested that targeting CX3CL1/CX3CR1 signalling could be considered a useful way to delay the progression of AD by increasing neuronal Aβ clearance and reducing Aβ levels [61]. Li et al., performed their experiments using adenovirus-mediated gene transduction of BMSCs to deliver exogenous CX3CL1 and Wnt3a in APP/PS1 mice [62]. While CX3CL1 alone did not improve cognitive function, MSCs carrying CX3CL1 and Wnt3a ameliorated memory and learning [62]. Importantly, Li’s experiment demonstrated a direct link between cognitive functions and microglial neurotoxicity, confirming that microglia status (baseline or quiescent, activated and primed) sharply affects brain function in health and diseases, and this status is influenced upstream by CX3CL1/CX3CR1 signals. This all gives strength to the hypothesis that baseline quiescent microglia can influence per se the destiny of healthy and AD people in many conditions, such as ageing, and through various mechanisms, including the FKN pathway [63]. Finneran et al. [64] isolated the chemokine domain and mucin-like stalk fragment of CX3CL1 and cloned it into a pTR2-MCS vector to gain mutant tau Tg45 mice. The authors evaluated the impact of soluble CX3CL1 expression on cognition in tau rTg450 mice after the onset of cognitive deficits by intraparenchymal injections of FKN in the ventricular system through adeno-associated virus serotype 4. They documented that soluble FKN overexpression significantly improved cognitive performance but did not reduce tau hyperphosphorylation. Opposite findings were achieved by Bolos et al., who demonstrated that CX3CR1 deficiency was associated with impaired uptake and degradation of tau by microglia, although it should be noted that CX3CR knockout mice were used in Bolos’ experiments instead of mutant tau Tg45 mice. In addition, an interesting mechanism was highlighted by Bolos et al., consisting of a competition between tau protein and the natural ligand of CX3CR1, FKN. Using affinity chromatography, the authors showed that the amount of tau internalization in vitro is reduced in the presence of CX3CL1, highlighting that microglia strongly influence tau internalization via CX3CR1 [59]. However, this event happens at the cost of disturbing the signalling of CX3CL1 and causing the activation of microglia [59]. Actually, Nash et al., had previously reported overexpression of FKN in AD mouse models. In their experiments, APP/PS1 and Tg4510 mice were treated with adeno-associated virus serotype 9 expressing FKN along with a viral control vector expressing green fluorescent protein (GFP) instead of CX3CR1.

Results showed decreased tau pathology in Tg4510 mice and no changes in Aβ deposition in APP/PS1 mice treated with FKN. Based on these findings, the authors, with enthusiasm, proposed CX3CR1 as a good target for AD treatment [65]. To address the issue of different activity and signals between the soluble and membrane-bound isoforms of CX3CL1, Lee et al., performed in 2014 an experiment on CX3CL1-deficient APPPS1 mice and crossed them with a mouse line expressing only soluble CX3CL1. Findings revealed that soluble CX3CL1 signalling does not influence fibrillar Aβ deposition and tau phosphorylation, while the membrane-bound isoform reduces Aβ and increases tau phosphorylation, being responsible for AD hallmark-related changes in CX3CL1 deficient mice. In addition, the authors observed enhanced phagocytosis and activation of microglia, postulating that the absence of membrane-bound FKN causes the reduction in Aβ deposition thanks to the p38 mitogen-activated protein kinase (MAPK)-mediated activation of microglia and the macrophage scavenger receptor 1 (MSR-1)-dependent phagocytosis [66].

In 2018, Bemiller et al., evaluated the effects of expressing only the chemokine domain of CX3CL1 on tau pathology and behavioural function. The authors used a mouse line expressing soluble FKN presenting only the chemokine domain of CX3CL1, crossed with tau pathology mouse models of hTau. Results showed that the chemokine domain of CX3CL1 fails to decrease both tau pathology and microglial activation [67], suggesting that this fragment of Cx3CL1 downregulates CX3CR1 expression by microglia and increases tau pathology. In addition, the authors confirm the hypothesis that neuron-microglia crosstalk mediated by CX3CL1/CX3CR1 signalling can cause, when dysfunctional or disrupted, the quiescent baseline status of microglia, giving rise to a phenotype of these cells, known as the primed phenotype, that loses protective ability toward neurons, acquires aggressive and neurotoxic behaviour and, in turn, underlies neuron damage and loss [63].

Even considering the above-mentioned flaws of these studies, it could be sound to hypothesize that a promising target for AD treatment could be searched for within the CX3CL1 signalling pathway and, generally, among molecules influencing microglia phenotypes.

## 3. Conclusions

A link between CX3CL1/CX3CR1 signalling, microglial phenotypes and neuronal damage and loss has been long suggested, with a growing body of data around the possible role of the FKN pathway in AD pathogenesis. Despite the many advances and insights on this topic that have been gained, unique findings in the literature are lacking, mainly due to flaws in the studies performed. However, some fixed points on this topic to be used as valuable hints for future investigations have been established, including the following: (i) CX3CL1/CX3CR1 signalling determines how microglia respond to stimuli, with Aβ deposition being a stimulus; (ii) chronic activation of microglia and their shift toward neurotoxic phenotypes is associated with the onset and progression of AD, with FKN having a crucial role in the phenotype switching of these cells; (iii) FKN regulates synaptic plasticity and enhances the neurogenesis of the adult at the site of the hippocampus, favouring learning and memory process, which is strongly linked to the pathogenesis of AD.

Molecules and transducers involved, respectively, in the upstream and downstream CX3CL1/CX3CR1 signalling cascade, such as p38 and metalloproteinases, represent a major target for modulating microglia activation, inflammation in the brain and neuronal protection through neuron-microglia communication. A valuable molecular target for AD treatment should be sought within those molecules capable of limiting and attenuating microglia priming and brain inflammation. However, studies to confirm a possible role for FKN in AD treatment are requested to be aligned as per the animals used, the protein fragment analyzed (soluble versus membrane-bound) and the concentrations used, considering that dose-dependent effects have been observed among the experiments.

## Figures and Tables

**Table 1:** Known downstream transducers of FKN signal and their expressions increase or decrease upon CX3CL1 stimulus. These transducers help convey signals modulating microglia activation, prevent priming and brain inflammation and enhance hippocampus neurogenesis. These mechanisms are all directly involved in AD pathogenesis, representing valuable targets for AD treatment. NF-kB: nuclear factor kB; MSK1: mitogen- and stress-activated kinase 1; Akt: protein kinase B; MSR-1: macrophage scavenger receptor 1; TNF-α: tumor necrosis factor α; TGF-β: transforming growth factor-β; Smad2: Caenorhabditis elegans Sma genes and the Drosophila Mothers against decapentaplegic proteins (Mad); NRF2: nuclear factor E2-related factor 2; NFE2L2: nuclear factor erythroid 2-like 2; P38MAPK: p38 mitogen-activated protein kinase; NLRP3: nucleotide-binding and oligomerization domain-like receptors (NLRs) and pyrin domain-containing protein 3 (P3); iNOS: Inducible nitric oxide synthase.

Downstream Transducer	Effect
✓ Il-1β ✓ IL-1α	↓ ↑
✓ NF-kB ✓ MSK1	↑ ↑
✓ Akt ✓ CCL2	↑ ↓
✓ MSR-1 ✓ IL-6 ✓ TNF-α ✓ TGF-β/Smad2 ✓ NRF2/NFE2L2 ✓ P38MAPK ✓ NLRP3 ✓ iNOS	↑ ↓ ↓ ↑ ↑ ↑ ↑ ↑

**Table 2:** List of FKN-related brain activities influencing cognitive functions such as learning and memory and behavioural functions such as stress resilience, anxiety and mood regulation. LTP: long term potentiation.

Brain Activities Depending on FKN Signalling
✓ synaptic pruning, maturation, and electrophysiological properties ✓ motor learning ✓ hippocampus neurogenesis
✓ spatial learning ✓ motor learning ✓ associative learning
✓ LTP

## Data Availability

All data supporting reported results and conclusions can be found in pubmed.com.
